# Configuration paths of community cafe to enhance residents’ well-being: fsQCA analysis of 20 cases in Shanghai

**DOI:** 10.3389/fpubh.2023.1147126

**Published:** 2023-06-16

**Authors:** Man Zhang, Tao Shen, Yongqi Lou

**Affiliations:** College of Design and Innovation, Tongji University, Shanghai, China

**Keywords:** community public space, social interaction, residents’ well-being, community cafe, fsQCA, configuration path

## Abstract

Community cafes have emerged as a critical infrastructure for promoting communication and cultural construction in urban areas, and have gradually become an essential public place to enhance residents’ well-being. However, despite their growing importance, more empirical research is needed on the emerging concept of community cafes, including the configuration analysis of their influencing factors. To address this gap, this study employs the fuzzy set qualitative comparative analysis (fsQCA) method to examine 20 community cafes in Shanghai, China. The configuration effects on residents’ well-being are explored across five dimensions: activity quality, psychological cognition, physical quality, physical accessibility, and sociability. The findings reveal that sociability is necessary for high levels of residents’ well-being. Three configuration paths are identified to generate high well-being, which can be classified into activity-based or acquaintance-based social interaction patterns based on spatial functions. Additionally, the study identifies five groups of non-high well-being configurations, in which lack of activity quality and sociability are core conditions. Overall, the study contributes to evaluating community public spaces and provides insight into the configuration of factors that contribute to residents’ well-being. The study highlights that community public spaces can have significantly different impacts on residents’ well-being, with sociability emerging as a significant factor. Therefore, it is necessary to clarify community public spaces’ social orientation according to spatial conditions.

## 1. Introduction

Community cafes have become increasingly popular in recent years, as more people recognize their value in enhancing residents’ well-being (1–3). In the research on public space, cafes as common consumption and leisure places in cities have received extensive attention from urban studies and social research. The intersection between cafes as physical spaces and their social-spatial significance has received particular attention (4–6). According to Oldenburg (7), cafes are a typical “third place” where people are bound by social norms but not totally ruled by society. In cafes, people are more likely to form social connections and encourage social interaction. Cafes have gradually become an important infrastructure for promoting communication, cultural construction, and carrying public life in the city (8–10). Unlike chain coffee shops, community cafes are located in communities and primarily serve the leisure needs of local population. Community cafes have developed into a unique cultural space for the production and expression of social relations, with diverse social groups conducting social activities in these spaces, which further exemplifies Hamas’s concept of public space as a public sphere (11).

Urban planning has long been prioritized to improve the life quality and well-being of people. There are strong evidences that the urban-built environment has a profound impact on public health and well-being. For example, studies have found that access to public spaces can reduce stress and anxiety, increase physical activity, and improve social connections (12–14). An increasing number of scholars recognize the value of public space in enhancing urban vitality, strengthening residents’ sense of belonging, and promoting their physical and mental health (15–17). In all scales of urban public space, the community and residents’ daily life are inseparable, as the basic unit and cell of urban function. The quality of the community environment directly impacts residents’ well-being (18–20). Therefore, community public spaces founded on commonality and neighborhood interaction are essential for preserving the social fabric of the neighborhood and enhancing residents’ well-being (21, 22).

The term “community public space” refers to a physical area within a community that is equally accessible to all its members. While it covers both physical and social space, this study focuses on the latter as community public spaces have a strong social significance, providing a variety of social roles as places for social interaction. These spaces are directly connected to residents’ daily life, which situate in the neighborhoods with strong interpersonal links and have a solid public nature. Scholars suggest that community public spaces play a crucial role in fostering community identity, promoting happiness, upholding neighborhood peace, and integrating social relationships (12–14). As a result, among the various forms of public spaces in communities, current studies particularly emphasize those that serve as places for social interaction, such as libraries, community centers, and different kinds of local shops that offer leisure and amusement activities. These places aim to strengthen cultural and communal ties in the neighborhood through specific spatial forms and public services (3, 23, 24).

From the perspective of public space, empirical research on community cafes is necessary. According to the 2022 China Ready-made Coffee Category Development Report released by Meituan (25), Shanghai ranks first in China in terms of the number of cafes, with community cafes taking up an important part, which provides this study with abundant local research objects. However, research on cafes has not yet identified the key factors that contribute to their significance as public spaces, especially for the emerging community cafes. The question is, what factors are the key to influencing the community cafe to enhance residents’ well-being?

This study aims to explore the crucial factors that influence community cafes’ ability to enhance residents’ well-being. Public space theory and interaction and space theory provide a theoretical basis for finding the impact factor of community cafes. Public space is viewed as a multifaceted concept encompassing social characteristics, public sphere significance, and physical environment quality. As it possesses both physical and social environment qualities, its effect on residents’ well-being is a complicated process involving several interrelated aspects. To clarify the multiple configuration paths of community cafes to enhance residents’ well-being, this study adopts the fuzzy set qualitative comparative analysis (fsQCA) to deal with these complex cause-and-effect relationships. This paper integrates the five antecedents of activity quality, including psychological cognition, physical quality, physical accessibility, and sociability through a literature review and exploratory factor analysis (EFA) to study the relationship among various configurations of these five antecedents and residents’ well-being. This paper attempts to answer the following questions: How do community cafes affect residents’ well-being? What are the configuration paths of influence? Which paths are the dominant ones?

## 2. Literature review

This paper investigates community cafes and their configuration paths as community public spaces to enhance residents’ well-being. This section aims to conduct theoretical modeling by combining the literature as the selection of condition variables. Although existing literature has studied public spaces, a gap exists in evaluating community-scale public spaces as a distinct research object. Typically, research on community-scale public space uses public space theory as a theoretical foundation and combines it with specific research questions. Given the emphasis on the social interaction attributes of community public spaces in this study, the theoretical modeling is mainly based on two theories: public space theory and interaction and space theory. Public space theory focuses on space’s physical and social properties. The former represents the physical environment quality of space, while the latter is the main focus of this article due to its relevance to the community and social attributes. Interaction and space theory, which draws from behavioral psychology and architectural behavior, is suitable for refining the theoretical model. Based on these two theories, this study constructs the initial evaluation dimensions of community public space. To further refine the evaluation dimensions and focus on the community scale, exploratory factor analysis will be conducted. This section will delve into the three subcategories of related literature, including the physical environment quality, political philosophy, and interaction &space perspectives.

### 2.1. Physical environment quality perspective

As a physical form, public space’s physical environment quality is the earliest influencing factor. Comfort, quality, and esthetic considerations have been identified as key variables for measuring the utilization of public spaces and are principally related to the physical and functional properties of the public space itself (26).

Comfort is considered one of the most important standards of public space (27), which is a subjective feeling of human beings to the physical space environment. It has an important influence on space behavior and is directly reflected in people’s usage of space. Quality of service and facilities is another important factor that affects people’s experience in public spaces and promotes social behavior (28, 29). The better people’s needs in public spaces are met, the higher the quality of service and facilities. This is critical for improving residents’ satisfaction (26). Furthermore, esthetics are important in attracting people’s attention and increasing their pleasure (30, 31).

Moreover, safety is a fundamental human need and is also taken into consideration when creating public spaces (32–34). According to Jacobs (35), safety is the fundamental principle of urban design. Safety is also the basic requirement in Maslow’s theory (36). As the basic unit of urban function, community is the closest to residents’ daily life and the concept of “home.” Therefore, safety is particularly important for community public space. Referring to the framework of Pikora et al. (30), safety means providing safe physical environments for residents.

Additionally, hygiene is critical for crowded spaces. Hence, cleanliness and tidiness are one of the basic conditions of public space and will impact how well a public space functions (37–39). Congestion level is also considered to be an important factor influencing the quality of public space, as spatial density can intuitively affect people’s experience in public space and hint at the allocation of public resources (26, 40).

### 2.2. Political philosophy perspective

Public space has deep roots in political philosophy due to its communal nature. Habib (41), a political scientist, summed up the public space theory into three main ideological models: H. Arendt’s philosophical view on the public realm, the liberals’ view on the legitimacy of power, and J. Habermas’s public sphere theory. In the political philosophy of the built environment, accessibility in public space is the most important. This concept was first put forward by historian S. Howard, who thinks that accessibility, which means “accessible to all,” is the foundation of the spatial entity of public space.

In summary, physical and psychological accessibility to public spaces are fundamental considerations for all public space planning (42). Many scholars have provided indicators of accessibility from these two aspects (43, 44). Physical accessibility is one of the most important indexes in evaluating public spaces (43–46). It refers to the effort made by residents to reach the public space from their starting point. The less effort required, the greater the physical accessibility (47). Psychological accessibility, on the other hand, focuses on the social nature of space, emphasizes the publicity and openness of space. It is also closer to Habermas’s concept of the public sphere. According to Bertolini, an accessible public space is one that different people can come to and do different things: it is both a node and a place (48, 49). From this perspective, accessibility means inclusiveness, which is regarded as a prerequisite for urban public space by sociologist L. Lofland (50).

### 2.3. Interaction and space perspective

Interaction is a fundamental aspect of human sociality, and in the context of urban planning, the behavior of individuals within a community is a basic unit in the structure of social communication. As the Machu Picchu Charter states: “We believe that human interaction and communication are the essential reasons for the city’s very existence. This reality must be reflected in urban planning and housing design (51).” The theory of interaction and space draws on environmental psychology and architectural behavior to regulate social interaction by examining the relationship between the environment and human psychological behavior. According to this theory, public space should focus on public life rather than just physical space, because human behavior is the most important factor in space (16).

One of the most widely recognized methods for evaluating the quality of urban public space and the public living conditions of citizens is called PSPL (Public Space and Public Life Survey) by Gehl (52). This approach evaluates both the physical quality of public space (PS) and the quality of behavior in space, which is the core of the approach (PL). The PS aspect represents the physical quality of the space, focusing on the protection and enjoyment of the space, with the former referring to people’s perceived safety in the space and the latter denoting the space’s potential to provide a positive sensory experience. The PL aspect evaluates the quality of behavior in space, as public space is the carrier of public activities, and the quality of activities determines whether people’s physiological and emotional needs can be met. Gehl (52) categorizes human activities in public space as “necessary activities” when participants have no choice, “optional activities” when people are willing to participate, and the spatial conditions are suitable, and “social activities” when actions rely on the participation of others. Among them, the latter two, known as “unnecessary activities,” are more easily affected by the quality of public space and can bring richer emotional experiences.

Similarly, Carr (53) emphasizes the centrality of human activity in public space, as the uniqueness of public spaces emerges from the various activities within them. He identifies five types of reasons for people’s needs in public spaces: comfort, relaxation, passive engagement, active engagement, and discovery (47). The first two causes are related to people’s perceived state of mind in public spaces. Comfort is the most basic need, as people feel their needs are met only when being comfortable. Relaxation is a more developed state of comfort in which both body and mind are at ease. The last three causes correspond to people’s behavior in public spaces, with varying degrees of initiative and desire when interacting with the space and others in it.

In addition, Oldenburg (7) introduces the concept of the “third place,” which refers to informal public gathering spaces outside of people’s homes and workplaces, such as cafes, bars, and community centers. The “third place” is often regarded as a “neutral zone,” with a high degree of inclusiveness and accessibility, allowing individuals to get psychological comfort and support while feeling comfortable. People can also engage in continuous dialog with each other in this space, making it a valuable forum.

However, it is worth noting that some scholars have raised concerns about the potential negative impact of excessive social interaction in public spaces on personal privacy, emphasizing the importance of prioritizing privacy in the design of public spaces (54–56). This can be achieved by increasing the physical distance between individuals or regulating the frequency and intensity of social interactions to allow for moments of solitude. Striking a balance between the public and private nature of public spaces is crucial, as inhibiting social interaction altogether may also have unintended consequences.

### 2.4. Hybrid perspective

In addition, some scholars have proposed comprehensive indicators for evaluating public spaces, drawing on the aforementioned perspectives. For example, Project for Public Spaces (PPS), a non-profit organization, has put forward four essential qualities that generate desired patterns of behavior, emotion, and measurable outcomes in public space after investigating more than 3,000 public spaces (53). These qualities include “social,” “have a variety of uses and activities,” “well-connected to their surroundings,” and “comfortable and welcome.” According to Erkip (26), factors affecting satisfaction of the users in public spaces are classified as accessibility, congestion levels, measures of comfort, the variety of activities and facilities. Indicators of quality, safety, physical attractiveness, or maintenance are classified as an esthetic consideration.

Based on the above literature, this paper constructs a list of impact factors of community cafes, as shown in Table 1.

**Table 1:** Evaluation factors of community cafes.

Number	Factor	Meaning	Definition	Literature
1	Comfort	The comfort feeling one perceives in the space.	A person’s perceived comfort level in the space.	Pasaogullari and Doratli (42)
			People feel comfortable subjectively in the physical environment.	Vukmirovic et al. (27)
			People’s mental state in the space.	Erkip (26)
			Get psychological comfort and support and feel comfortable.	Oldenburg and Brissett (7)
			The comprehensive feeling of people in the space.	Madden and Wiley-Schwartz (53)
			The mental state that one perceives in the space.	Carr (47)
2	Quality	The quality of services and facilities in the space.	The quality of service and facilities.	Pasaogullari and Doratli (42)
			Services and facilities can meet the needs of people in public spaces.	Erkip (26)
			Quality of services and infrastructure.	Stauskis (28)
				Stauskis and Eckardt (29)
3	Esthetic considerations	The physical space’s esthetic features and whether they are appealing to people.	Physical attractiveness and maintenance.	Pasaogullari and Doratli (42)
			Physical attractiveness.	Erkip (26)
			Esthetic features of the physical environment and facilities.	Pikora et al. (30)
			Human esthetic preference for physical space.	Matsuoka and Kaplan (31)
4	Safety	The safe feeling one perceives in the space.	Personal safety.	Navarrete-Hernandez et al. (32)
			The safety of life shall not be infringed.	Burton and Mitchell (33)
			Avoid fear and outside risks.	Van der Burgt (34)
			Psychological safety and physical safety.	Jacobs (35)
			Provide a safe physical environment for residents.	Pikora et al. (30)
			People can feel safe in the space.	Gehl (52)
5	Cleanliness	The sanitary condition of the space.	Sanitary conditions in public Spaces.	Carmona (37)
			Clean and well maintained spaces.	Beck (38)
			Cleanliness of environment and facilities.	Williams and Green (39)
6	Congestion level	Whether there is enough space to support people’s behaviors.	Spatial density.	Erkip (26)
			The coordination between spatial scale and behavior.	Gehl (52)
			Density of resource allocation in public space.	Webster (40)
7	Physical accessibility	The effort made by people to reach the space from their starting point.	Barrier-free experience in public places.	Brorsson et al. (44)
			The ease with which a building, place or facility can be reached by people and/or goods and services.	Lotfi and Koohsari (45)
			Distance and travel time.	Erkip (26)
			Whether it is easy to access public spaces.	Talen (43)
			Distance and time to public space.	Ward Thompson and Travlou (46)
8	Inclusiveness	People of different identities participate in the activities of space.	The public nature of space.	Bertolini (48)
			Public Spaces are accessible to different groups of people.	Bertolini and Dijst (49)
			Welcome to everyone.	Madden and Wiley-Schwartz (53)
			Open to all identities and groups.	Nadal (50)
			Everyone can come and go without restrictions.	Oldenburg and Brissett (7)
9	Activity	The variety of human activities in the space.	The activities that people can carry out in public space can be divided into necessary activities, optional activities and social activities.	Gehl (52)
			A variety of uses and activities.	Madden and Wiley-Schwartz (53)
			The variety of activities and facilities.	Erkip (26)
			People’s initiative and desire when interacting with space and others in space can be divided into three levels: passive engagement, active engagement, and discovery.	Carr (47)
10	Social interaction	Interact with other people in the space.	Engage in a heart-to-heart and ongoing conversation.	Oldenburg and Brissett (7)
			Interaction with others.	Madden and Wiley-Schwartz (53)
			Social activities that require interaction with other people.	Gehl (52)
11	Enjoyment	People’s pleasure level in the space.	People keep a happy mood in the space.	Askari and Soltani (57)
			People feel ease and relaxation in the space.	Carr (47)
12	Privacy	Protect personal privacy in public Spaces.	Personal privacy is not subject to random inspection.	Easwara Moorthy and Vu Kim-Phuong (54)
			Personal information will not be disclosed.	Little et al. (55)
			Not to be disturbed by others.	Herzfeld (56)

## 3. Exploratory factor analysis

Based on the literature review, a total of 12 evaluation factors were identified and summarized in Table 1. To determine the condition variables, an exploratory factor analysis was conducted to remove any factors that were not relevant to the study object. Specifically, 113 residents and community-building scholars were inquired with questionnaires to assess the importance of factors in improving the well-being of residents in community cafes. The above 12 influencing factors were scored by Likert’s five levels. KMO is 0.710, and the data passed the Bartlett test (*p* < 0.05), indicating that the data is suitable for factor analysis, as shown in Table 2.

**Table 2:** KMO and Bartlett’s test.

KMO		0.710
Bartlett test	Approx. Chi-Square	638.940
	df	66
	*p* value	0

Factor analysis extracted five dimensions, with the variance rate after rotation being 27.002, 22.462, 12.832, 9.044, and 8.374%, respectively, and the cumulative rate is 79.714%, as shown in Table 3.

**Table 3:** Total variance explained.

Factor	Eigen		% of Variance (Unrotated)			% of Variance (Rotated)			
	Eigen	% of Variance	Cumulative % of variance	Eigen	% of Variance	Cumulative % of variance	Eigen	% of Variance	Cumulative % of Variance
1	3.24	27.002	27.002	3.24	27.002	27.002	2.684	22.37	22.37
2	2.695	22.462	49.464	2.695	22.462	49.464	2.48	20.668	43.038
3	1.54	12.832	62.296	1.54	12.832	62.296	1.695	14.128	57.166
4	1.085	9.044	71.34	1.085	9.044	71.34	1.689	14.072	71.239
5	1.005	8.374	79.714	1.005	8.374	79.714	1.017	8.475	79.714
6	0.809	6.739	86.452	–	–	–	–	–	–
7	0.445	3.711	90.164	–	–	–	–	–	–
8	0.371	3.093	93.257	–	–	–	–	–	–
9	0.319	2.658	95.914	–	–	–	–	–	–
10	0.201	1.674	97.589	–	–	–	–	–	–
11	0.166	1.38	98.969	–	–	–	–	–	–
12	0.124	1.031	100	–	–	–	–	–	–

As shown in Table 4, the relationship between each factor and the item is analyzed through factor loading. Variable 12 was excluded because its factor loading is less than 0.5. Finally, five factors, namely activity quality, psychological cognition, physical quality, physical accessibility, and sociability, are selected as condition variables, as shown in Table 5.

**Table 4:** Factor loading (rotated).

Number	Factor loading					Communality
	Factor 1	Factor 2	Factor 3	Factor 4	Factor 5	
1	**0.888**	0.213	−0.028	−0.023	0.049	0.838
4	**0.872**	0.167	−0.099	−0.239	−0.002	0.855
11	**0.887**	0.141	−0.156	−0.034	−0.072	0.838
3	0.04	**0.874**	0.064	0.239	0.067	0.831
5	0.143	**0.927**	0.07	0.098	0.002	0.895
6	0.246	**0.769**	0.022	0.099	0.02	0.662
2	−0.078	0.032	**0.879**	0.033	−0.039	0.782
9	−0.169	0.178	**0.817**	0.062	−0.041	0.733
8	−0.039	0.184	0.026	**0.885**	−0.094	0.828
10	−0.158	0.172	0.089	**0.873**	0.091	0.832
7	0.002	0.066	−0.042	−0.009	**0.983**	0.972
12	0.452	−0.267	0.449	0.067	0.136	0.5

Bold values represent factor loads greater than 0.5.

**Table 5:** The outcome of exploratory factor analysis.

Factor	Item number	Name
Activity quality	2	Quality
	9	Activity
Psychological cognition	1	Comfort
	4	Safety
	11	Enjoyment
Physical quality	3	Esthetic considerations
	5	Cleanliness
	6	Congestion level
Physical accessibility	7	Physical accessibility
Sociability	8	Inclusiveness
	10	Social interaction

### 3.1. Activity quality

The quality of activities in a community public space is determined by various behaviors of individuals in the space. The higher the diversity and initiative of these activities, the better the quality. Additionally, the availability of services and facilities in the space plays a significant role in facilitating and enhancing the quality of activities. To measure “activity,” the proportion of unnecessary activities in the public space is taken as the specific measurement index. A questionnaire is used to gather information on residents’ activities, indicating their willingness and ability to participate in these activities. When measuring “quality,” the specific measurement index is the score of 7-level Likert questionnaires on residents’ satisfaction with services and facilities in public spaces.

### 3.2. Psychological cognition

The psychological impact of space on individuals is profound, and the emotions perceived by people in space will directly affect people’s behavior in space and evaluation of space. When people feel “comfort,” “safe,” and “enjoyment” in the space, they will not reject the experience in the space. Since all three indicators are subjective, residents will use the Likert 7-level scale to answer how much they perceive these three mental states in the space.

### 3.3. Physical quality

Since community public space is first and foremost a physical space of a physical entity, its spatial quality directly impacts residents’ well-being. “Esthetic considerations” refer to the physical space’s esthetic features and whether they appeal to residents. “Cleanliness” refers to whether the environment of community public space is clean and tidy. “Congestion level” refers to whether there is enough space to support people’s behaviors and activities. The specific measurement indicators of the above three items are all scored by the 7-level Likert questionnaire.

### 3.4. Physical accessibility

According to the relevant literature, “physical accessibility” means the convenience of residents to access the community public space. The specific measurement index is the travel time from the place of residence to the public space.

### 3.5. Sociability

The sociability of community public space implies a wide range of social interactions between people of various identities. Community public space is more than just a physical environment for residents to meet their needs; it is also a public domain open to all residents to promote communication, understanding, and integration. As a result, community public space should not be restricted to a specific demographic. “Inclusiveness” means that people of different identities participate in the activities of community public space. The specific measurement index is what residents score on the 7-level Likert questionnaire in the field of “inclusiveness.” At the heart of “social interaction” is a conversation between people (7); the more vibrant the conversation in public space, the more likely people interact socially. The specific measurement index is the proportion of residents who engage in conversation in public spaces.

**Figure 1:**
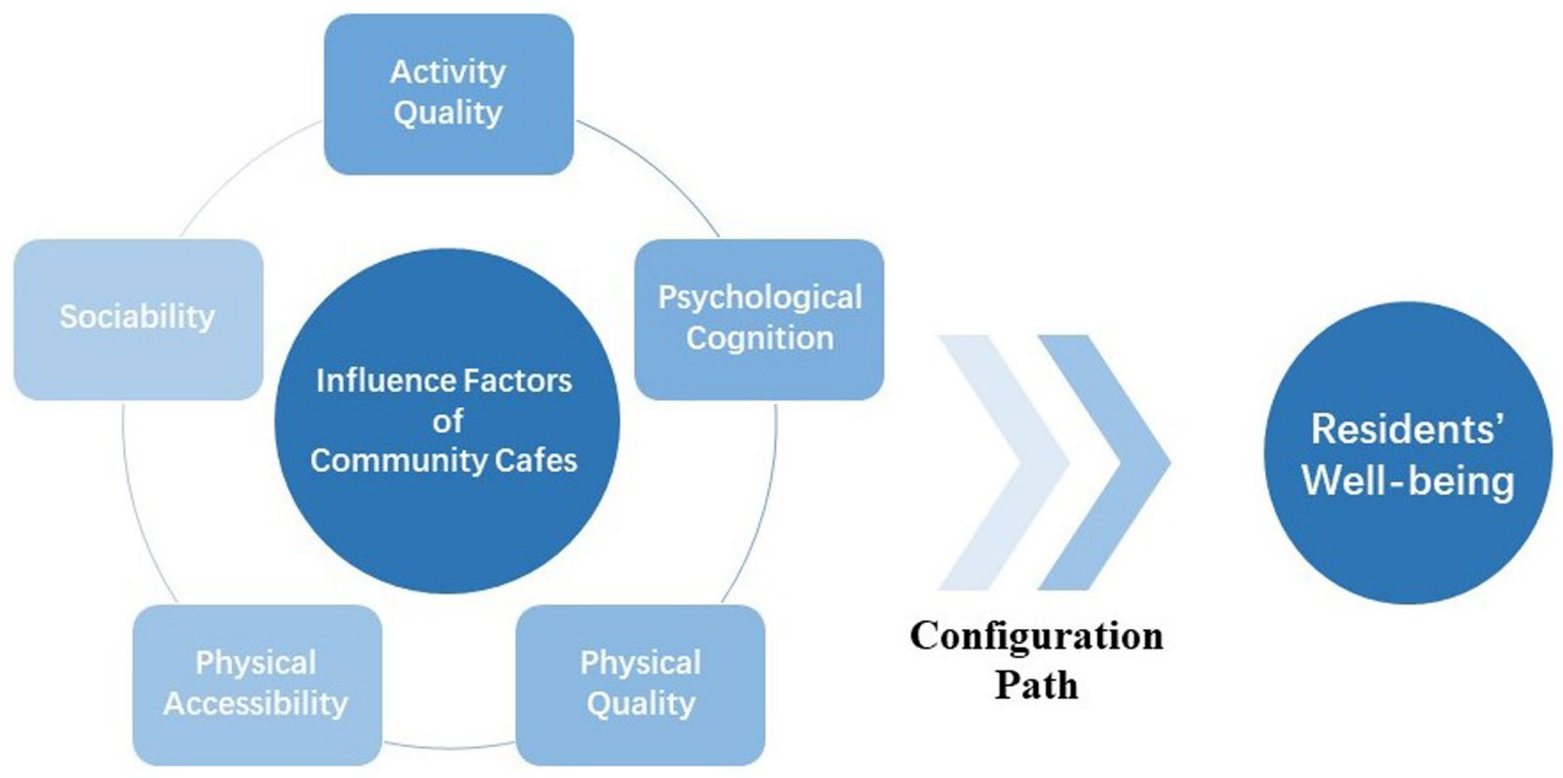
The relationship between condition variables and the outcome variable. Five interrelated condition variables form different configurations, so as to produce diverse paths for community cafes to affect resident’s well-being.

In summary, the relationship between condition variables and the outcome variable is shown in Figure 1.

## 4. Materials and methods

### 4.1. fsQCA

In the 1980s (58), American sociologist Ragin proposed a case-oriented asymmetric research method called qualitative comparative analysis (QCA) to solve complex causal phenomena. QCA, based on set theory and Boolean operation, investigates how the configuration of antecedent conditions affects the interpretation of results. Compared to traditional causal inquiry methods, this method addresses issues such as causal asymmetry, multiple concurrent causal relationships, etc. Through consistency and coverage, QCA primarily evaluates the relationship between condition and outcome variables. Consistency refers to the extent to which cases with specific outcome variables share the configuration of a particular group of condition variables. When the consistency is more significant than 0.8, it shows that the configuration constitutes a sufficient condition for a specific result. It is necessary when the value is more significant than 0.9 (59).

The configuration of community cafes affecting residents’ well-being is a complex process influenced by multiple factors. Because each factor is not isolated from the others, it is appropriate to use the QCA method to deal with complex causal problems. Since the condition variables are continuous variables representing degree, they are not ideal for clear set comparative analysis (csQCA) and multivalued set comparative analysis (mvQCA). Instead, this study uses fuzzy set qualitative comparative analysis (fsQCA) to transform all data into membership degrees from 0 to 1. This paper analyzes the influence of activity quality, psychological cognition, physical quality, physical accessibility, and sociability on residents’ well-being using a sample of 20 community cafes in Shanghai, China.

### 4.2. Variable selection

The definitions and indicators of condition variables have been described in the previous section. The outcome variable needs to measure the well-being of residents.

From the philosophical notion of utility perspective, Diener and other scholars put forward that subjective well-being is one of the main methods to evaluate the quality of social life, with the other two being economic and social indicators (60). Subjective well-being includes cognitive experience and emotional experience, which is an important index to measure the quality of personal and social life (61–63). This paper adopts the Index of well-being, developed by Campbell et al. (64), and it is a mature and widely used well-being scale (62).

The scale consists of two parts, the first part is an index of general affect, including eight items, and the second part is an index of life satisfaction, which has only one item. When calculating the total score, the average score of the total general affect is added (weighted 1) with the life satisfaction score (weighted 1.1). The score ranges between 2.1 (the lowest well-being) and 14.7 (the highest well-being). Based on theoretical modeling and exploratory factor analysis, the final variables and their measures are shown in Table 6.

**Table 6:** Explanation and measurement of variables.

Variable type	Name	Secondary indicator	Explanation	Measurement
Condition variable	Activity quality	Quality	The quality of services and facilities in the space.	7-level Likert questionnaire for residents’ sense of satisfaction with services and facilities in public space.
		Activity	The variety of human activities in the space.	The proportion of the types of unnecessary activities that people can do in public spaces to all types of activities.
	Psychological cognition	Comfort	Subjective feeling when people are in the space.	7-level Likert questionnaire for residents’ sense of these three mental states in the space.
		Safety		
		Enjoyment		
	Physical quality	Esthetic considerations	The physical space’s esthetic features and whether they are appealing to residents	7-level Likert questionnaire for residents’ sense of these three factors.
		Cleanliness	The sanitary condition of the space.	
		Congestion level	Whether there is enough space to support people’s behaviors and activities.	
	Physical accessibility	Physical accessibility	The convenience of residents to the community public space.	The time from the place of residence to the public space.
	Sociability	Inclusiveness	People of different identities participate in the activities of space.	7-level Likert questionnaire for residents’ sense of inclusiveness.
		Social interaction	Interact with other people in the space. At the heart of “social interaction” is conversation between people.	The proportion of residents who engage in conversation in public spaces.
Outcome variable	Subjective well-being (SWB)	-	A subjective feeling includes both cognitive experience and emotional experience.	Campbell’s index of well-being.

### 4.3. Data

Based on field investigation and the information from dianping.com, a third-party consumer review website, this paper selects 20 community cafes situated in non-commercial residential areas across Shanghai, China, as representative research samples, as shown in Table 7. These cafes have been widely discussed and praised for their community attributes on dianping.com. Through field investigation and online contact, a total of 206 questionnaires were distributed to the nearby residents who had visited these cafes. The questionnaire topics were designed according to the specific measurement indicators detailed in Table 6. The secondary indicators’ scores are added to the condition variables’ scores, and the final scores of the 20 cases were obtained by computing the average scores of the questionnaires. Finally, the score table of 5 condition variables and 1 outcome variable was obtained, as shown in Table 8.

**Table 7:** Sample source and basic information.

Sample number	Name (abbreviations for privacy reasons)	Located community	Third-party consumer review website score (out of 5)
1	BA Cafe	Jiashan community	4.5
2	m Coffee	YOU + International Youth Community	4.2
3	p	Anshan fifth village	4.5
4	R Coffee	Yan Heng Riverside Garden	3.7
5	P1 Coffee	Jialanting	4.3
6	CL Coffee	Guoquan Road 333 Lane Community	4.7
7	W	Maoming South Road 163 Community	4.6
8	S1	Zhong He Apartment	4.2
9	B Cafe	SVA Expo Garden	3.8
10	P2 Coffee	West New Villa	4.0
11	TH Coffee	Beimengsan Community	4.8
12	LD Coffee	Zhengdan East Road Community	4.7
13	YY Coffee	Xiangyang South Road 510 Lane Community	4.6
14	AL Coffee	Xinyi Yayuan	4.3
15	ASC Coffee	Zhongshan Xintun	4.6
16	CA	Ruijin New Village	4.8
17	CBS	Julu Road 272 Lane Community	4.5
18	CS	No.2 Community, Lane 838, Beijing West Road	4.5
19	S2	Urumqi Road 148 Lane Community	4.5
20	M Coffee	Xinhu Qinglan International Community	3.9

**Table 8:** Variables’ original scores.

Sample	Condition variable					Outcome variable
	Activity quality	Psychological cognition	Physical quality	Physical accessibility	Sociability	SWB
1	6.68	17.93	14.67	4.5	3.6	9.89
2	7.03	19.31	19.33	5.56	7.11	11.85
3	6.77	18.7	18.5	5.75	7.42	11.25
4	6.5	18.08	18.49	5.67	7.23	12.38
5	7.64	17.24	16.86	6.14	7.86	13.35
6	7.21	18.86	14.71	6.43	7.29	13.2
7	7.35	19.13	19.1	4.18	7.27	11.86
8	7.21	17.16	15.64	6.75	7.75	13.43
9	7.21	19	18.7	3.7	7.1	12.66
10	6.87	17.32	17.82	4.45	6.73	12.47
11	7.12	18.19	18.96	4.24	7.08	13.07
12	7.17	18.23	18.5	4.23	7.05	13.11
13	7.16	18.74	18.67	4.76	6.96	12.49
14	7.2	17.95	18	4	7	12.33
15	7.4	18.93	18	3.4	7.6	13.41
16	7.41	19.72	19.57	4.83	7.24	13.98
17	7.75	20.1	20.4	2.8	7.4	14.28
18	7.65	19.45	19.16	2.17	8	14.23
19	7.3	18.6	18	5	7.8	13
20	6.88	19.08	18	3.8	7.2	13.16

### 4.4. Variable calibration

To convert the original data into set membership scores, the process of assigning set membership scores to cases and conditions is called calibration (65). The direct calibration method is used in this study. Combined with the numerical characteristics and referring to the commonly used QCA calibration anchors (66, 67), 95% quantile, 50% quantile, and 5% quantile are set as calibration anchors in this paper, which represents, respectively, full membership, the crossover point, and full non-membership, as shown in Table 9. Specific calibration results are shown in Table 10.

**Table 9:** Calibration anchors.

Variable type	Variable name	Calibration		
		Full membership	Crossover point	Full non-membership
Condition variable	Activity quality	7.75	7.21	6.5
	Psychological cognition	20.081	18.72	17.164
	Physical quality	20.3585	18.495	14.672
	Physical accessibility	6.734	4.475	2.2015
	Sociability	7.993	7.235	3.7565
Outcome variable	SWB	14.2775	13.035	9.958

**Table 10:** Calibration values.

Sample	Condition variable					Outcome variable
	Activity quality	Psychological cognition	Physical quality	Physical accessibility	Sociability	SWB
1	0.09	0.18	0.05	0.51	0.04	0.04
2	0.32	0.79	0.79	0.81	0.47	0.24
3	0.13	0.49	0.5	0.84	0.68	0.15
4	0.05	0.23	0.5	0.83	0.5	0.35
5	0.92	0.05	0.22	0.9	0.92	0.68
6	0.51	0.58	0.05	0.93	0.55	0.6
7	0.69	0.71	0.73	0.4	0.53	0.24
8	0.51	0.05	0.1	0.95	0.88	0.72
9	0.51	0.65	0.58	0.26	0.47	0.41
10	0.19	0.06	0.37	0.49	0.39	0.37
11	0.41	0.26	0.68	0.42	0.47	0.52
12	0.46	0.28	0.5	0.42	0.46	0.55
13	0.45	0.51	0.57	0.59	0.44	0.37
14	0.49	0.18	0.4	0.35	0.45	0.33
15	0.75	0.61	0.4	0.19	0.81	0.71
16	0.76	0.9	0.85	0.62	0.5	0.91
17	0.95	0.95	0.96	0.1	0.66	0.95
18	0.92	0.83	0.74	0.05	0.95	0.95
19	0.63	0.44	0.4	0.67	0.9	0.49
20	0.2	0.69	0.4	0.29	0.49	0.57

After calibration, a case with a 0.5 membership degree appeared. This is not included in the analysis because it’s difficult to classify, which affects the final analysis results. Therefore, referring to the research of Fiss (59) and Wagemann et al. (68), all membershi*p* values less than 1 are increased by 0.001 in actual operation.

## 5. Results

In this study, fsQCA3.0 software is used to analyze the configuration path of improving residents’ well-being in 20 community cafes. According to the suggestions of Fiss (59), Greckhamer et al. (69), and An et al. (70), the parameters are set: the original consistency threshold is 0.8, the PRI consistency threshold is 0.5, and the case frequency threshold is 1.

### 5.1. Necessity analysis of single conditions

The first step of QCA analysis is to analyze the necessity of single conditions, that is, to check whether the result set is a subset of a certain condition. The criterion of necessary condition analysis is that the consistency is higher than 0.9. As can be seen from Table 11, the necessity of sociability exceeds 0.9, which constitutes a necessary condition. The other condition variables do not constitute necessary conditions, which shows that the explanatory power of each single condition variable to the outcome variable is weak. Therefore, the configuration analysis of these condition variables will be carried out below.

**Table 11:** Analysis of necessary conditions.

	High SWB		Non-high SWB	
Condition variable	Consistency	Coverage	Consistency	Coverage
Activity quality	0.847591	0.865462	0.607528	0.599598
~ Activity quality	0.607866	0.615737	0.863683	0.845618
Psychological cognition	0.733530	0.788583	0.620956	0.645243
~ Psychological cognition	0.670010	0.646490	0.796541	0.742884
Physical quality	0.752212	0.779816	0.716785	0.718247
~ Physical quality	0.728220	0.726791	0.780265	0.752699
Physical accessibility	0.683382	0.653195	0.800814	0.739849
~ Physical accessibility	0.727827	0.790812	0.624619	0.655983
Sociability	0.904621	0.794473	0.743642	0.631261
~ Sociability	0.580138	0.700713	0.757884	0.884798

### 5.2. Configuration analysis

fsQCA determines the intermediate and parsimonious solutions, as well as the core and marginal conditions. The core conditions that co-occur in the intermediate and parsimonious solutions significantly impact the results. Conditions that appear only in the intermediate solution are marginal.

The results of the QCA analysis are shown in Table 12, in which three configuration paths produce high SWB. The consistency indexes of the two configurations are 0.924585，0.913148, and 0.945302, respectively. The overall solution consistency is 0.904417, all of which are higher than 0.9, which shows that both configurations are sufficient conditions for high well-being. The overall solution coverage is 0.785251, indicating that the three configurations explain about 79% of the reasons for high well-being.

**Table 12:** Configuration results.

	Solution							
Configuration	High well-being			Non-high well-being				
	1	2	3	4	5	6	7	8
Activity quality	●	●	●	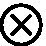	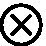	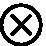	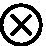	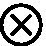
Psychological cognition	●	●			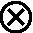	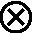	●	●
Physical quality		●	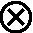	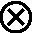		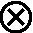	●	●
Physical accessibility		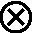	●	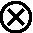	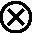		●	●
Sociability	●		●	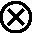	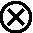	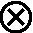	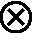	●
Consistency	0.9246	0.913148	0.945302	0.96325	0.97076	0.987159	0.963091	0.997641
Raw coverage	0.6028	0.523107	0.530187	0.506613	0.506612	0.625636	0.398169	0.516378
Unique coverage	0.0446	0.0234022	0.159095	0.0193285	0.00590026	0.0791454	0.0417091	0.0463885
Overall solution consistency	0.904417			0.949743				
Overall solution coverage	0.785251			0.753611				

● and ● mean the conditions exist. 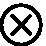 and 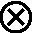 mean the conditions do not exist. ● and 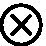 denote the core conditions. ● and 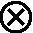 denote the marginal conditions. White space denotes that the condition may or may not exist.

**Figure 2:**
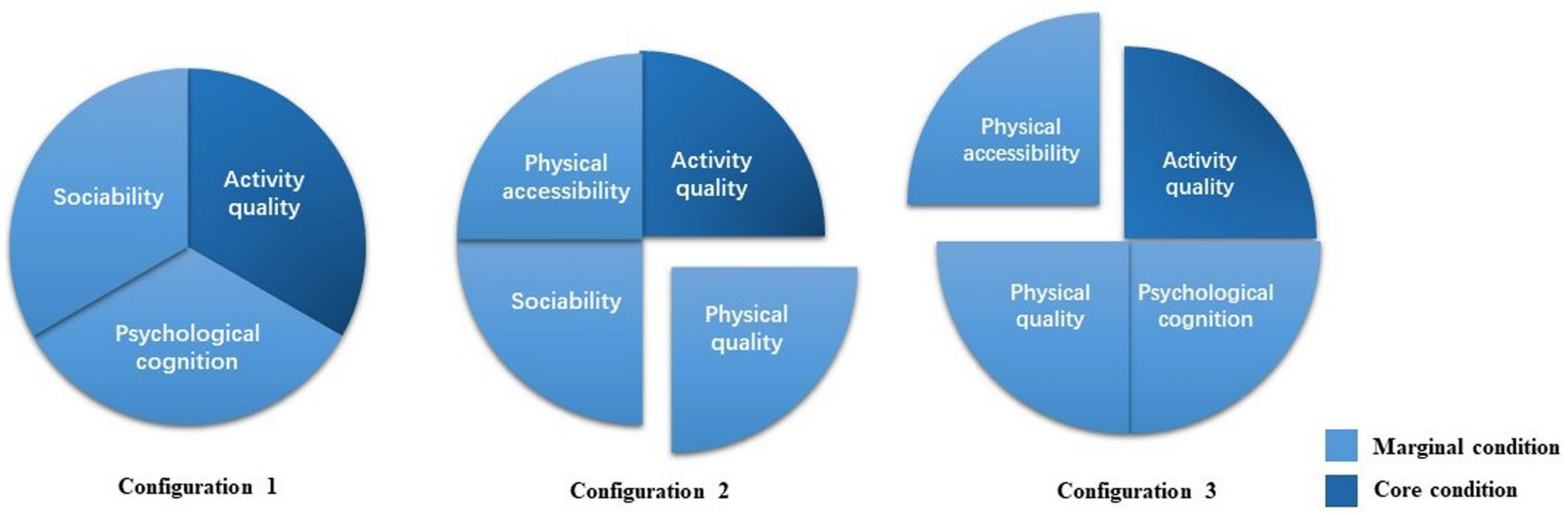
Configuration paths of community public space with high well-being. QCA results show three configuration paths producing high resident well-being. The core condition of configuration 1 is high activity quality, the marginal conditions include high sociability and high psychological cognition. The core condition of configuration 2 is high activity quality, the marginal conditions include high sociability, high physical accessibility, and non-high physical quality. The core condition of configuration 3 is high activity quality, the marginal conditions include high psychological cognition, high physical quality and non-high physical accessibility.

Meanwhile, considering the asymmetry of cause and effect, the reasons for the appearance and non-appearance of results are different, such that the conditions for opposite results must be examined separately. Because the condition variables and the outcome variable selected in the theoretical modeling part are positively correlated, it is assumed that the absence of any condition variable may lead to a lower level of well-being. The data is then subjected to another fsQCA operation. The results show that five configuration paths produce non-high well-being, that their consistencies are more significant than 0.9, and that the overall solution coverage is 0.753611, indicating that these five paths provide sufficient conditions for non-high well-being and explain approximately 75% of the causes. According to the findings of the analysis, this study creates a configuration path model diagram of community public space to improve the well-being of residents (Figure 2).

(1) Activity-based space: configuration 2. Its core conditions are high activity quality, and the marginal conditions are high psychological cognition, high physical quality, and low physical accessibility. This suggests that, regardless of the sociability of the community cafe, residents will have a higher sense of well-being if other conditions are met, even if they have less convenience and take longer to reach. Activities in this type of space go beyond simply selling coffee and may include workshops, reading clubs, parent–child activities, pet activities, and more. Residents in this type of space can engage in various unnecessary activities that can provide new and pleasant psychological experiences. These activities help to stimulate diverse psychological experiences and create a solid social atmosphere (52). Furthermore, the larger spatial scale of this kind of community cafe in all samples allows it to accommodate more residents and pay more attention to decoration and space design, resulting in higher physical space quality. This demonstrates that high physical space quality helps improve the experience of social communication spaces (71). It is worth noting that due to the large space and high rent, this kind of community cafe is relatively remote and has poor physical accessibility. However, the other excellent qualities compensate for this flaw.
(2) Acquaintance-based space: configuration 1 and 3. Both have high activity quality as their core condition and high sociability as their marginal condition. Still, configuration 1 has high psychological cognition, while configuration 3 has high physical accessibility and low physical quality. This difference is due to the different groups that each space serves. Configuration 1 consists of themed cafes that attract a specific group of people through a unique feature and therefore has no strict requirements for the quality and accessibility of the physical space. It mainly serves as a place for small groups to meet, focusing on people’s psychological feelings and interaction within the space. On the other hand, configuration 3 has a small spatial scale in general and primarily sells high-quality coffee while also providing a small space for nearby residents to have social conversations. Due to space constraints, these cafes do not have enough room for large events, and they tend to be crowded and do not pay much attention to store decoration. However, residents often gather here to socialize, supporting Hall’s (72) conclusion that the distance between people identifies social relations in public space. As people’s connections increase, the distance between them shrinks, and intimate crowding develops in public spaces. The nearby regular residents are the leading consumer group of this type of cafe, which is related to the higher physical accessibility. The easier for users to access a specific space, the higher the usage rate (52).

By comparing these two types, we found that both of them have high activity quality, but the main difference lies in their social function. The former is mainly activity-based, while the latter is primarily acquaintance-based. This further demonstrates the importance of community cafes as “the third space” (7). Furthermore, according to the coverage index, configurations 1 and 3 have slightly higher scores than configuration 2, indicating that they are more likely to improve residents’ well-being. This fully demonstrates that for community public spaces, the physical attributes of the space itself are less important than the social attributes (73), and the deep interaction between people is the key to improving residents’ well-being.

(3) The core conditions of configurations 4, 5, 6, and 7 are characterized by non-high activity quality, and the marginal conditions are also non-high sociability. While configurations 4, 5, and 6 lack positive conditions, configuration 7 has all the other positive conditions. This indicates that, regardless of other factors, a community cafe lacking high activity quality and sociability will inhibit residents’ well-being. This further underscores the significance of activity quality and sociability in community public spaces.

Cases in configurations 4, 5, and 6 mainly consist of small shops that only provide coffee sales services. After customers drink coffee, they typically leave without engaging in other activities, resulting in an indifferent atmosphere. Additionally, some shops are located in remote areas that are challenging to spot. These community cafes often result in poor business and low foot traffic.

In contrast, cases in configuration 7 mainly consist of medium to large cafes that are situated near residential areas and have high-quality physical space. However, residents typically drink coffee or spend time alone without interacting with others. Configuration 8, while possessing all other positive conditions, lacks activity quality. This finding further emphasizes the importance of the behavioral perspective in enhancing residents’ well-being. Referring to the original data, it was found that the sociability score of the configuration 8 cases had a higher inclusiveness score, which may explain the difference between configurations 8 and 7. Nonetheless, the cases in both configurations were similar in terms of lacking social interaction.

QCA causality is asymmetric, implying that different condition configurations are required to explain the occurrence of a result. Compared to the previous five configurations, the study revealed that the lack of activity quality and sociability in community cafes has a noticeable inhibitory effect on residents’ well-being. The study also uncovered that psychological cognition, physical quality, and physical accessibility play a substitute role in explaining non-high well-being.

### 5.3. Robustness analysis

To verify the robustness of the analysis results, this study adjusted the consistency threshold according to the practice of White et al. (74) and changed the consistency standard from 0.8 to 0.85 and 0.72, respectively. The configuration path and parameters did not change substantially and passed the robustness test.

## 6. Discussion

The main results are as follows. Firstly, through theoretical modeling and exploratory factor analysis, it is found that community cafes have an important impact on residents’ well-being through five aspects: activity quality, psychological cognition, physical quality, physical accessibility, and sociability. There are three main ways to produce high well-being, which demonstrates the multiple causal relationships of this impact.

Secondly, an analysis of the necessity of individual conditions reveals that sociability is crucial for promoting well-being. This underscores the emphasis placed in the existing literature on the social value attribute of public spaces, which distinguishes them from other types of urban spaces (7, 75, 76). From this perspective, community public spaces play a critical role in urban development, not only in terms of their physical layout within the urban environment but also in the positive experiences that people derive from their use of these spaces (52). When a space functions as a “social space,” its design must move beyond the mere transformation of the objective physical environment and consider environmental studies, behavior, psychology, and sociology to ensure that people have a positive social experience in the built environment (77). This underscores the importance of community public spaces as shared communication spaces for public life (47). As Arendt (78) argues, the shared nature of these spaces is an essential attribute of public life and is key to consolidating and maintaining community consciousness. Thirdly, configuration analysis has revealed that the three configurations associated with high well-being can be distinguished by differences in their social function. The activity-based configuration requires sufficient activity venues and environmental quality. The acquaintance-based configurations emphasize high sociability but ignore the physical form quality of the space and distinguish their primary users based on accessibility. Fourthly, asymmetric analysis reveals that five configurations produce low well-being. Among the five condition variables, lack of activity quality and sociability are the main conditions that make it difficult for community cafes to improve residents’ well-being.

### 6.1. Theoretical contribution

This study integrates five key factors from public space theory and interaction & space theory, comprising a total of 11 variables, to investigate the impact of community cafes, as typical community public spaces, on residents’ well-being. Previous studies have focused on limited factors, and the internal mechanism of the synergistic influence of the comprehensive elements of the five factors remains unclear. Additionally, few studies have proposed evaluation indicators for community-scale public spaces. Therefore, this study enriches the evaluation dimension of community public space, and conducts a deep analysis of the influence of factor configuration on residents’ well-being. Based on the literature review and exploratory factor analysis, this study establishes a relatively comprehensive theoretical framework and explores the complex concurrent causal relationship between community cafes and residents’ well-being, which is beneficial in revealing the black box of this causal relationship and providing specific theoretical support for the construction practice of community public space. In addition, this study also demonstrates the complex configuration relationship among influencing factors, suggesting that researchers and practitioners should not only focus on single critical conditions but also consider the configuration effect among different conditions. Moreover, through the QCA method, this study demonstrates a causal asymmetry of the impact, indicating that the paths to high and non-high well-being are not entirely opposite. The negative reasons cannot be analyzed simply by opposing the influence paths of high well-being.

### 6.2. Practical enlightenment

#### 6.2.1. Strengthen the social attribute of community public space

This study supports the importance of the social aspects of community public space. It warns the community construction industry, which has recently overemphasized physical spatial attributes while ignoring social attributes. Scholars generally believe that the social significance of the built environment is as important as the apparent meaning of spatial intention, and its social value for public space is even more in line with the connotation of “public” (37, 48, 50). From a semiotic perspective, the built environment reflects the cultural characteristics of society, and its meaning changes as social values change (79). The environmental significance of community public space lies in building the cultural identity of the community, especially because of its social orientation. Communities are not only the most minor functional division in an urban environment, but they are also widely regarded as the more important basic units of actual and potential solidarity and social cohesion. With the geographical construction of neighborhoods and a range of possible expectations, the community has been endowed with community-like expectations for security and connection, which has become a complex spatial form integrating identity, use, and action (80).

As one of the earliest scholars who introduced the term “public space” into urban research, Jacob (35) believes that public space is critical in promoting good social interaction in community building. The value of public space lies in the social understanding and integration brought by its inclusiveness and diversity, which is an important source to enhance residents’ well-being. Scholars generally believe that social interaction in public spaces is a vital link to maintaining social relations at different levels. It serves as a balance and supplement to the private sphere (29, 73, 81–83).In fact, different communities based on geographical background have gradually evolved different social characteristics, which may lead to systematic community differences in residents’ well-being (84). Under the background of rapid urbanization, the connection between neighborhood and community has cracked, leading to various “community questions” (85). This demonstrates the principle that must be addressed in community building: returning to the concept of community, treating the community as viable units of identity and action, and paying attention to the community’s sociality and intimacy. Community public space is a critical breaking point in the modern high-density and high-privacy housing development trend.

#### 6.2.2. Clarify the social orientation of community public space

According to configuration analysis, this study suggests that the community public space should flexibly choose the mode of activity-based or acquaintance-based according to the type of activity, spatial scale, and geographical location. This also demonstrates that the space environment can express and transmit meaning through various elements, leading to the difference in human behavior (77). Activity-based public space confirms the significance of space in shaping people’s behavior, emphasized by the theory of communication space (52). It also illustrates the role of complex space in promoting social interaction (53). This reveals that community public space with good social attributes needs to stimulate residents’ spontaneous and social activities through specific physical space elements. The acquaintance-based community public space more obviously reflects the different attitudes of different groups toward the community interpersonal network. According to Relph (86), different people can give meaning to space through life experiences, and the key to transforming space into place is a sense of belonging. This sense of belonging comes not only from the experience triggered by the physical form of space, but also from the human network built based on space. The main users of this kind pay attention to smaller, closer, and more frequent interpersonal networks. They tend to have more spatially proximate and robust neighbor networks (87). Therefore, it is easier to form tight but selective social ties (88). This type of social tie implies the integration of social cohesion and positive social control over public space, which is called collective efficiency and helps to reduce unsafe factors such as disorder and crime, giving people a good psychological experience (89).

#### 6.2.3. Avoid extremes and improve the conditions of community public space according to the actual situation

Since the reasons affecting residents’ well-being are asymmetric, the causes of non-high well-being cannot be reversed according to the reasons of high well-being, demonstrating that the opposites of configurations 1,2, and 3 cannot be considered as lessons to inhibit residents’ well-being. Similarly, we cannot assume that improving the factors of configurations 4, 5, 6,7, and 8 will improve the residents’ well-being. The development of community public space needs to combine the actual situation and comprehensively consider various antecedent conditions.

## 7. Conclusion and research limitation

This study aims to investigate the complex configuration paths of community cafes to enhance residents’ well-being. Therefore, the fuzzy set qualitative comparative analysis (fsQCA) method was adopted to analyze 20 typical cases in Shanghai, China, as samples. The condition variables were selected from 12 factors identified in the literature and reduced to 11 factors and five dimensions through exploratory factor analysis. The outcome variable is subjective well-being. This study constructs a theoretical framework that explains how community cafes influence residents’ well-being through activity quality, psychological cognition, physical quality, physical accessibility, and sociability. The results indicate that the impact has significant configuration differences, among which the sociability condition is particularly important. The current data highlight the importance of clarifying community public space’s social orientation according to spatial conditions.

This study appears to be one of the first attempts to examine the configurations of community-scaled public space’s effect on residents’ well-being. The insights gained from this study may be of assistance to community-building research and practice. However, it is important to note that the study has some limitations, including limited sample size and coverage. Furthermore, the investigation focuses on exploring the relationship between condition variables and the outcome variable, and it does not analyze the influencing factors of condition variables in detail. Future research could further explore larger-scaled community public space samples and delve deeper into the condition variables with greater granularity.

## Data availability statement

The original contributions presented in the study are included in the article/Supplementary material, further inquiries can be directed to the corresponding author.

## Ethics statement

Ethical review and approval was not required for the study on human participants in accordance with the local legislation and institutional requirements.

## Author contributions

YL led the overall study. MZ and TS jointly completed the research plan and manuscript writing. ZM was also responsible for data collection and analysis. All authors read, contributed to the research design, and approved the final manuscript.

## Funding

The authors acknowledge the funding support for this work received from Tongji University “Innovative Design and Intelligent Manufacturing” Discipline Cluster Project (F2201).

## Conflict of interest

The authors declare that the research was conducted in the absence of any commercial or financial relationships that could be construed as a potential conflict of interest.

## Publisher’s note

All claims expressed in this article are solely those of the authors and do not necessarily represent those of their affiliated organizations, or those of the publisher, the editors and the reviewers. Any product that may be evaluated in this article, or claim that may be made by its manufacturer, is not guaranteed or endorsed by the publisher.
