# Supplementary material for: Configuration paths of community cafe to enhance residents’ well-being: fsQCA analysis of 20 cases in Shanghai
Source: Front Public Health. 2023 Jun 16;11:1147126. doi: 10.3389/fpubh.2023.1147126 (PMC10314294; doi:10.3389/fpubh.2023.1147126)
Supplement: Supplementary file 1 [file Table_1.docx]

Supplementary Material

# Questionnaire Design

Please rate your feelings in this cafe, ranging from 1 to 7. The higher the score, the more you agree.

A It's very comfortable.

B I feel safe here.

C The inclusiveness here is very high (open to any group and class).

D The space environment design here is very attractive.

F I feel enjoyment here.

Please rate the following contents of this cafe, ranging from 1 to 7. The higher the score, the more you agree.

A It's clean and tidy here.

B There is plenty of space here and it is not crowded.

C I am very satisfied with the service and facilities here.

Please check, what activities have you done here?

Drink coffee or eat

stay with friends

Reading

Do nothing, rest, be in a daze

Work or study

Participate in special activities, workshops, etc. in the store

Meet strangers and chat with them

Photographing

Others: Please fill in the blanks

Please check it. What is the time from your place of residence to this cafe?

More than one and a half hours

1 hour 15 minutes-1 and a half hours

1 hour-1 hour 15 minutes

45-1 hour

30-45 minutes

15-30 minutes

Within 15 minutes

The following two items are Index of Well-being.

5.1 The general affect you feel in your life, representing the degree from 1 to 7.

Interesting 1 2 3 4 5 6 7 boring

enjoyable    1 2 3 4 5 6 7 miserable

valuable    1 2 3 4 5 6 7 useless

friendly   1 2 3 4 5 6 7 lonely

fulfilling     1 2 3 4 5 6 7 empty

hopeful 1 2 3 4 5 6 7 hopeless

rewarding    1 2 3 4 5 6 7 disappointing

Life is good to me   1 2 3 4 5 6 7 life gives me no chance

5.2 How satisfied or dissatisfied are you with your life in general? Which value is closest to your satisfaction or dissatisfaction?

Very dissatisfied      1 2 3 4 5 6 7 very satisfied

# Original Data of the Questionnaire

| Condition variable | Activity  quality | | | Psychological  cognition | | | | Physical  quality | | | | Physical  accessibility | Sociability | | | Subjective  well-being |
| --- | --- | --- | --- | --- | --- | --- | --- | --- | --- | --- | --- | --- | --- | --- | --- | --- |
| Secondary indicator | Quality | Activity | Total  score | Comfort | Safety | Enjoyment | Total  score | Aesthetic considerations | Cleanliness | Congestion level | Total  score | - | Inclusiveness | Social interaction | Total  score | - |
| 1 | 6 | 0.68 | 6.68 | 6.5 | 5 | 6.43 | 17.9 | 5.67 | 3 | 6 | 14.67 | 4.5 | 2.9 | 0.7 | 3.6 | 9.89 |
| 2 | 6.33 | 0.7 | 7.03 | 6.44 | 6.44 | 6.43 | 19.3 | 6.33 | 6.44 | 6.56 | 19.33 | 5.56 | 6.67 | 0.44 | 7.11 | 11.85 |
| 3 | 6 | 0.77 | 6.77 | 6 | 6.5 | 6.2 | 18.7 | 7 | 5 | 6.5 | 18.5 | 5.75 | 6.67 | 0.75 | 7.42 | 11.25 |
| 4 | 5.83 | 0.67 | 6.5 | 5.33 | 6.5 | 6.25 | 18.1 | 5.83 | 6.33 | 6.33 | 18.49 | 5.67 | 6.56 | 0.67 | 7.23 | 12.38 |
| 5 | 6.86 | 0.78 | 7.64 | 5.14 | 6.43 | 5.67 | 17.2 | 6.43 | 5.57 | 4.86 | 16.86 | 6.14 | 6.86 | 1 | 7.86 | 13.35 |
| 6 | 6.57 | 0.64 | 7.21 | 5.57 | 6.86 | 6.43 | 18.9 | 5.71 | 4 | 5 | 14.71 | 6.43 | 6.43 | 0.86 | 7.29 | 13.2 |
| 7 | 6.64 | 0.71 | 7.35 | 6.36 | 6.27 | 6.5 | 19.1 | 6.64 | 5.82 | 6.64 | 19.1 | 4.18 | 6.45 | 0.82 | 7.27 | 11.86 |
| 8 | 6.5 | 0.71 | 7.21 | 4.63 | 6.75 | 5.78 | 17.2 | 5.88 | 3.88 | 5.88 | 15.64 | 6.75 | 6.75 | 1 | 7.75 | 13.43 |
| 9 | 6.4 | 0.81 | 7.21 | 6.4 | 6.1 | 6.5 | 19 | 6.4 | 6 | 6.3 | 18.7 | 3.7 | 6.4 | 0.7 | 7.1 | 12.66 |
| 10 | 6.14 | 0.73 | 6.87 | 5.82 | 5.64 | 5.86 | 17.3 | 5.91 | 5.77 | 6.14 | 17.82 | 4.45 | 5.91 | 0.82 | 6.73 | 12.47 |
| 11 | 6.4 | 0.72 | 7.12 | 6.36 | 6.2 | 5.63 | 18.2 | 6.36 | 6.04 | 6.56 | 18.96 | 4.24 | 6.32 | 0.76 | 7.08 | 13.07 |
| 12 | 6.41 | 0.76 | 7.17 | 6.14 | 6.09 | 6 | 18.2 | 6.5 | 5.86 | 6.14 | 18.5 | 4.23 | 6.23 | 0.82 | 7.05 | 13.11 |
| 13 | 6.48 | 0.68 | 7.16 | 6.14 | 6.1 | 6.5 | 18.7 | 6.43 | 6 | 6.24 | 18.67 | 4.76 | 6.29 | 0.67 | 6.96 | 12.49 |
| 14 | 6.5 | 0.7 | 7.2 | 6.25 | 5.5 | 6.2 | 18 | 6.25 | 5.25 | 6.5 | 18 | 4 | 6.5 | 0.5 | 7 | 12.33 |
| 15 | 6.6 | 0.8 | 7.4 | 6.6 | 6 | 6.33 | 18.9 | 6.2 | 5.4 | 6.4 | 18 | 3.4 | 6.8 | 0.8 | 7.6 | 13.41 |
| 16 | 6.71 | 0.7 | 7.41 | 6.57 | 6.71 | 6.44 | 19.7 | 6.29 | 6.57 | 6.71 | 19.57 | 4.83 | 6.57 | 0.67 | 7.24 | 13.98 |
| 17 | 7 | 0.75 | 7.75 | 6.8 | 6.8 | 6.5 | 20.1 | 7 | 6.8 | 6.6 | 20.4 | 2.8 | 6.4 | 1 | 7.4 | 14.28 |
| 18 | 6.83 | 0.82 | 7.65 | 6.67 | 6.33 | 6.45 | 19.5 | 6.83 | 6 | 6.33 | 19.16 | 2.17 | 7 | 1 | 8 | 14.23 |
| 19 | 6.4 | 0.9 | 7.3 | 6 | 6.4 | 6.2 | 18.6 | 6.2 | 5.4 | 6.4 | 18 | 5 | 6.8 | 1 | 7.8 | 13 |
| 20 | 6.2 | 0.68 | 6.88 | 6.4 | 6.2 | 6.48 | 19.1 | 6.2 | 6.2 | 5.6 | 18 | 3.8 | 6.2 | 1 | 7.2 | 13.16 |
